# Validation of MTL30 as a quality indicator for colorectal surgery

**DOI:** 10.1371/journal.pone.0238473

**Published:** 2020-08-28

**Authors:** Niels Matthes, Johannes Diers, Nicolas Schlegel, Mohammed Hankir, Imme Haubitz, Christoph-Thomas Germer, Armin Wiegering

**Affiliations:** 1 Department of General, Visceral, Transplantation, Vascular and Pediatric Surgery, University Hospital Wuerzburg, Wuerzburg, Germany; 2 Comprehensive Cancer Center Mainfranken, University Hospital Wuerzburg, Wuerzburg, Germany; 3 Department of Biochemistry and Molecular Biology, Theodor Boveri Institute, University of Wuerzburg, Wuerzburg, Germany; UKSH Campus Lübeck, GERMANY

## Abstract

**Background:**

Valid indicators are required to measure surgical quality. These ideally should be sensitive and selective while being easy to understand and adjust. We propose here the MTL30 quality indicator which takes into account 30-day mortality, transfer within 30 days, and a length of stay of 30 days as composite markers of an uneventful operative/postoperative course.

**Methods:**

Patients documented in the StuDoQ|Colon and StuDoQ|Rectal carcinoma register of the German Society for General and Visceral Surgery (DGAV) were analyzed with regard to the effects of patient and tumor-related risk factors as well as postoperative complications on the MTL30.

**Results:**

In univariate analysis, the MTL30 correlated significantly with patient and tumor-related risk factors such as ASA score (p<0.001), age (p<0.001), or UICC stage (p<0.001). There was a high sensitivity for the postoperative occurrence of complications such as re-operations (p<0.001) or subsequent bleeding (p<0.001), as well as a significant correlation with the CDC classification (p<0.001). In multivariate analysis, patient-related risk factors and postoperative complications significantly increased the odds ratio for a positive MTL30. A negative MTL30 showed a high specify for an uneventful operative and postoperative course.

**Conclusion:**

The MTL30 is a valid indicator of colorectal surgical quality.

## Background

Surgery is often the best and only therapeutic option available for a large number of diseases but comes with specific complication rates which increase with procedural complexity [1]. Treatment quality and the adequate management of complications arising from surgery are therefore critical factors for therapeutic success [2]. Numerous studies have shown that complication rates largely depend on patient-specific factors such as age or the ASA score on the one hand, and on the infrastructure of the treating hospital and its expertise for specific procedures on the other [3–7].

The quality of a given medical therapy can broadly be divided into 3 parts: The quality of the structure, which reflects the available equipment at a medical facility; the quality of the process, which reflects the way a medical treatment is carried out; and the quality of the result, which reflects the actual outcome of the treatment [8]. Quality indicators (QI) in surgery are measuring units which in general enable a graded distinction between good and bad operative/postoperative outcomes. They do not measure quality directly, but serve as surrogate parameters. A large number of parameters for visceral surgery have been described including 30- or 90-day mortality, postoperative length of stay, minor or major complications, anastomotic leakage, wound healing disorders, number of removed lymph nodes, quality of mesocolic excision, intraoperative blood loss, postoperative ventilation duration, resumption rate, and quality of life indices, which all could be used hypothetically to evaluate quality [9–11]. However, these indicators are influenced by a variety of factors, both patient-related (age, comorbidities) and the care system (availability of diagnostic and intervention options). They also require detailed and conscientious documentation. Ultimately, it is difficult to apply one of the QI listed above to a variety of operations in hospitals with different equipment.

An optimal QI should be applicable to all operations if possible, should be sensitive to the occurrence of complications, and should specifically depict comorbidities. In addition, it should be adjustable and take into account complex care structure across differently equipped hospitals.

We previously proposed the MTL30 as a general performance marker for surgical quality within the 30 day postoperative period [12]. In summary, if the patient died in inpatient care, MLT rated positive (M), whereas if they lived but are still in inpatient care at day 30, MLT rated positive (L). Further, if the patient was transferred to another acute care hospital (not for rehabilitation), MLT rated positive (T). Thus, the L and T designations reflect the occurrence of a major complication that either led to a significant extension of stay (L) or that could not be treated in the corresponding hospital (T). This allows adjustment between hospitals across different care levels. The time interval of 30 days was chosen for all designations to ensure comparability with the 30-day lethality. As it has been shown previously that daily documentation of complications is challenging, it is important to have access to pre-existing data [13]. The parameters that contribute to the MTL30 can be easily obtained from the hospital billing data which are universally documented and in contrast to the company's own documentations, are difficult or impossible to modify.

The German Society of General and Visceral Surgery (DGAV) have previously produced a large registry for patients to assess and to improve the quality of surgical treatment [14]. This includes the collection of data on the course of surgical treatment in hospitals in Germany. Importantly, all hospitals contribute to this registry, irrespective of their size or volume. This effectively reduces a potential selection bias, such as would occur if only high volume centers were included for instance, and enables the evaluation of quality markers over a wide range of patient groups and hospital categories.

Based on this registry, we conducted an analysis to characterize the MTL30 on colon and rectal cancer (StuDoQ|Colon Cancer; StuDoQ|Rectal Cancer registers) with respect to patient-specific factors and the sensitivity to complications.

## Methods

The StuDoQ|Colon carcinoma and StuDoQ|Rectal carcinoma registers are prospectively documented databases for surgical interventions in colorectal carcinomas, which were set up by the DGAV in January 2010 (www.dgav.de/studoq, www.en.studoq.de). They were developed to facilitate the assessment of the quality and risk factors of colorectal cancer surgery in Germany. The declaration of consent, ethic approvment and the data security procedures were approved by the Society for Technology, Methods and Infrastructure for Networked Medical Research (http://www.tmf-ev.de). Written consent was obtained from all participants. The publication guidelines were determined by the DGAV (http://www.dgav.de/studoq/datenschutzkonzept-und-publikations guidelines.html). The data from participating centers are entered prospectively in pseudonymized form using a browser-based tool and subjected to automatic plausibility checks. Validation by cross-checking with institutional medical control data is part of the annual certification process. For the present study, all cases that underwent curative resection due to colorectal cancer were identified in the StuDoQ|Colon cancer and StuDoQ|Rectal cancer registry, and relevant demographic data, comorbidities, as well as information on operations, histology and perioperative history for analysis extracted in anonymous form. Full wall excision, simple polypectomy, endoscopic mucosal resection, and other endoluminal procedures, as well as palliative interventions regardless of the size of the operation, were excluded. Basic registration structures are comparable to the StuDoQ|Pancreas registration [14].

Postoperative complications included anastomotic leakage (grade C) [15, 16], infection of the surgical site [17], Clavien-Dindo classification (CDC) [18], burst abdomen, reoperation, and hospital mortality. They were defined as either present or not present. Additional postoperative parameters that were assessed were the need for unscheduled postoperative ventilation lasting more than 48 hours, pneumonia, length of stay (LOS), and readmission. Postoperative total morbidity was summarized as none (CDC 0), minor (CDC 1–2), severe (CDC 3a-4), and fatal (CDC 5) according to the CDC. Patients were counted as MTL30 positive if they had died within 30 days after index operation, the postoperative length of stay exceeded 30 days or if they had been transferred to another acute hospital or in hospital unit (e.g. transfer to a tertiary center due to surgical complications or to internal medicine due to postoperative pulmonary emboly). Transfer to a postoperative rehabilitation did not count as positive.

Statistical analysis was performed with a bilateral significance level of 0.05. Scale variables were expressed as median and range and categorical parameters as absolute frequency and percentage. Univariate analysis was performed using the chi-square test for categorical variables and the Mann-Whitney test for ratio variables.

All variables with a p-value <0.1 in the univariate analysis were included in the multivariate analysis. The multivariable analysis was carried out by logistic regression.

## Results

A total of 19,646 patients diagnosed with colorectal cancer (66.6% colon resections, 33.4% rectal resections) were included in the evaluation. 55.85% (10,973) of the patients were male, the mean age at the time of surgery was 69.5 ± 11.9 years and mean BMI was 26 ± 4.96 kg/m^2^. Of all patients, 88.5% (17,930) were assigned to the ASA classification II / III. Tumor stages were UICC I 28.1% (4,963), UICC II 31.1% (5,485), UICC III 28.4% (5,000) and UICC IV12.4% (2,192), respectively. MTL30 positive were 9.59% (1,884) of all patients. For a detailed description of the patient population, see S1 Table.

In the univariate analysis, a positive MTL30 correlated with patient-specific pre-existing diseases. Accordingly, a positive MTL30 for patients with chronic obstructive pulmonary disease (8.94% vs. 20.19%; p <0.001), peripheral arterial occlusive disease (9.32% vs. 19.28; p <0.001), diabetes mellitus (NIDDM / IDDM) (8.72% vs. 11.48% / 16.12%; p <0.001), liver cirrhosis (9.28% vs. 23.1%; p <0.001), antihypertensive medications (6.1% vs. 11.95%; p <0.001), corticosteroids (9.48% vs. 18.52%; p <0.001) or alcohol abuse (9.28 vs. 17.31%; p <0.001) were significantly more frequently observed compared to the cohort in which these factors were absent. In the case of gradable risk factors (NYHA, cerebrovascular events) or the ASA score, the percentage of MTL30 positive patients correlated highly with the respective grade of the risk factor (Table 1).

**Table 1:** Impact of patients characteristics on MTL30.

		MTL30				
		normal		divergent		
		n	%	n	%	p
ASA	1	1638	9,22	40	2,12	
	2	8887	50,04	526	27,92	
	3	6863	38,64	1114	59,13	
	4	366	2,06	198	10,51	
	5	7	0,04	6	0,32	<0.001
Weight loss	no	15780	88,97	1585	84,26	
	yes	1956	11,03	296	15,74	<0.001
Diabetes mellitus	no	14329	80,67	1369	72,66	
	NIDDM	2314	13,03	300	15,92	
	IDDM	1119	6,30	215	11,41	<0.001
Cerebrovascular events	no	16573	93,31	1650	87,58	
	yes, w/o deficit	747	4,21	122	6,48	
	yes, with deficit	442	2,49	112	5,94	<0.001
CAD	no	14790	83,27	1340	71,13	
	yes	2972	16,73	544	28,87	<0.001
NYHA	negative	14376	80,94	1211	64,28	
	positive	3386	19,06	673	35,72	<0.001
	0	14376	82,83	1211	67,92	
	1	866	4,99	106	5,95	
	2	1424	8,21	241	13,52	
	3	640	3,69	190	10,66	
	4	49	0,28	35	1,96	<0.001
Dialysis	no	17675	99,51	1846	97,98	
	yes	87	0,49	38	2,02	<0.001
COPD	no	16857	94,90	1655	87,85	
	yes	905	5,10	229	12,15	<0.001
PAOD	no	17335	97,60	1782	94,59	
	yes	427	2,40	102	5,41	<0.001
Blood pressure medication	no	7448	41,93	484	25,69	
	yes	10314	58,07	1400	74,31	<0.001
Radio-/Chemotherapy	no	15414	86,78	1649	87,53	
	Radiotherapy	153	0,86	28	1,49	
	Chemotherapy	165	0,93	25	1,33	
	combined	2030	11,43	182	9,66	0.0032
Corticoids	no	17564	98,89	1839	97,61	
	yes	198	1,11	45	2,39	<0.001
Immunosuppressants	no	17626	99,23	1872	99,36	
	yes	136	0,77	12	99,36	0.53
Anticoagulants	no	14596	82,18	1375	72,98	
	yes	3166	17,82	509	27,02	<0.001
Alcohol abuse	no	17133	96,50	1753	93,10	
	yes	621	3,50	130	6,90	<0.001
Liver cirrhosis	no	17403	98,29	1780	95,14	
	yes	303	1,71	91	4,86	<0.001

There was also a significant correlation between positive MTL30 and tumor-specific parameters. The MTL30 was significantly more frequently positive in patients presenting with distant metastases, lymph node metastases and locally advanced tumors (T4) (Table 2). There was also a significant correlation with the pathological resection status (R0 9.34% vs. R1 15.02% / R2 22.77%, p <0.001) and tumor-related malnutrition (9.13% vs. 13.14%; p <0.001).

**Table 2:** Impact of tumor characteristics on MTL30.

		MTL30				
		normal		divergent		
		n	%	n	%	p
pT	pT0	467	2,64	42	2,24	
	pT1 (Submucosa)	2100	11,86	134	7,15	
	pT2 (Muscularis)	3368	19,02	303	16,18	
	pT3 (Subserosa)	9304	52,55	988	52,75	
	pT4a (Serosa)	1704	9,62	249	13,29	
	pT4b (neighboring organs)	761	2,64	157	8,38	<0.001
pTsm	sm1	487	33,94	29	32,58	
	sm2	304	21,18	11	12,36	
	sm3	644	44,88	49	55,06	0.064
pN	pN0	10939	61,78	1098	58,65	
	pN1a	1940	10,96	174	9,29	
	pN1b	1929	10,89	229	12,23	
	pN1c	250	1,41	33	1,76	
	pN2a	1217	6,87	152	8,12	
	pN2b	1432	8,09	186	9,94	<0.001
M	M0	15544	87,64	1531	81,44	
	M1a	1435	8,09	187	9,95	
	M1b	757	4,27	162	8,62	<0.001
UICC	I	4963	28,13	410	21,96	
	II	5485	31,09	608	32,57	
	III	5000	28,34	500	26,78	
	IV	2192	12,43	349	18,69	<0.001
Liver metastasis	no	16200	91,50	1643	87,63	
	yes	1504	8,50	232	12,37	<0.001
Lung metastasis	no	17310	97,77	1798	95,89	
	yes	394	2,23	77	4,11	<0.001
PC	no	17222	97,28	1768	94,29	
	yes	482	2,72	107	5,71	<0.001
other metastases	no	17339	98,15	1807	96,58	
	yes, others	288	1,63	57	3,05	
	yes, several others	39	0,22	7	0,37	<0.001
Resections status	R0	17210	97,95	1772	96,04	
	R1 micorscopic	283	1,61	50	2,71	
	R2 macroscopic	78	0,44	23	1,25	<0.001

Focusing on a positive MTL30 as a function of postoperative complications, there was a significant correlation with both operative and nonoperative complications. The group of patients with postoperative burst abdomen, wound healing disorders, postoperative haemorrhaging, ileus, blood transfusion or re-operation each had a significantly higher percentage of positive MTL30 (Table 3). Similarly, patients with postoperative non-surgical complications, such as prolonged ventilation for more than 48 hours, renal failure, pneumonia, or thromboembolic events (pulmonary embolism, myocardial infarction, stroke) each showed a significantly increased percentage of positive MTL30. There was also a significant correlation between the percentage of positive MTL30 and the severity of the complication according to Clavien-Dindo (Tables 4).

**Table 3:** Impact of surgical complications on MTL30.

		MTL30				
		normal		divergent		
		n	%	n	%	p_c_
Dehiscence	no	17336	97,60	1571	83,39	
	yes	426	2,40	313	16,61	<0.001
Bleeding	no	17478	98,40	1773	94,11	
	yes	284	1,60	111	5,89	<0.001
Sacral infection	no	17642	99,32	1814	96,28	
	yes	120	0,68	70	3,72	<0.001
Ileus	no	17260	97,17	1688	89,60	
	yes	502	2,83	196	10,40	<0.001
Fistula	no	17710	99,71	1808	95,97	
	yes	52	0,29	76	4,03	<0.001
other surgical complication	no	16651	93,75	1379	73,20	<0.001
	yes	1111	6,25	505	26,80	<0.001
Bladder emptying disorder	none	5217	92,53	459	84,38	
(surgical consequence)	delayed—discharge w/o urine drainage	186	3,30	34	6,25	
	delayed—discharge with urine drainage	222	3,94	41	7,54	
	long-term bladder emptying disorder	13	0,23	10	1,84	<0.001
Anastomotic leakage	none	16904	95,17	1172	62,21	
	A	154	0,87	31	1,65	
	B/C	355	2,00	282	14,97	
	not classified	349	1,96	399	21,18	<0.001
Clavien-Dindo	none	12376	69,82	223	16,98	
	1	959	5,41	39	2,97	
	2	2127	12,00	154	11,73	
	3a	795	4,48	108	8,23	
	3b	1218	6,87	559	42,57	
	4a	251	1,42	230	17,52	
	4b	36	0,20	133	10,13	
	5	0	0,00	438	33,36	<0.001
bleeding requirering transfusion	no	17578	98,96	1652	87,69	
	yes	184	1,04	232	12,31	<0.001
SSI 30	no	16235	91,56	1255	66,76	
	yes	1497	8,44	625	33,24	<0.001
Re-operation	none	16441	92,56	845	44,85	
	one time	1010	5,69	450	23,89	
	2-30x	311	1,75	589	31,26	<0.001

**Table 4:** Impact of non-surgical complications on MTL30.

		MTL30				
		normal		divergent		
		n	%	n	%	p_c_
Ventilation > 48h	no	17631	99,26	1408	74,73	
	yes	131	0,74	476	25,27	<0.001
Renal insufficiency	no	17722	99,77	1670	88,64	
	yes	40	0,23	214	11,36	<0.001
Lung embolism	no	17709	99,70	1817	96,44	
	yes	53	0,30	67	3,56	<0.001
Pneumonia	no	17358	97,73	1480	78,56	
	yes	404	2,27	404	21,44	<0.001
Stroke	no	17738	99,86	1845	97,93	
	yes	24	0,14	39	2,07	<0.001
Myocardial infarction	no	17719	99,76	1821	96,66	
	yes	43	0,24	63	3,34	<0.001
other non-operative complication	no	16151	90,93	1181	62,69	
	yes	1611	9,07	703	37,31	<0.001

The sensitivity that a positive MTL30 detects postoperative complication was increased with the grade of complication. While patients with a postoperative CDC 1 complication showed a positive MTL30 in 23.6% of all cases, this continuously increased to 94,1% of patients suffering from a CDC 4b complication (CDC 2: 26,8%; CDC 3a: 40,0%; CDC 3b: 47,5%; CDC 4a: 73,6%; CDC 5: 100,0%). On the other side, specificity for a negative MTL30 was 98.2% for patients without any complications, and slight decrease to 93.1% for CDC ≥ 4b. Also, specificity of a negative MTL30 was high to rule out occurrence of specific complication as AL (93,5%) or need for re-operation (95,1%).

**Fig 1:**
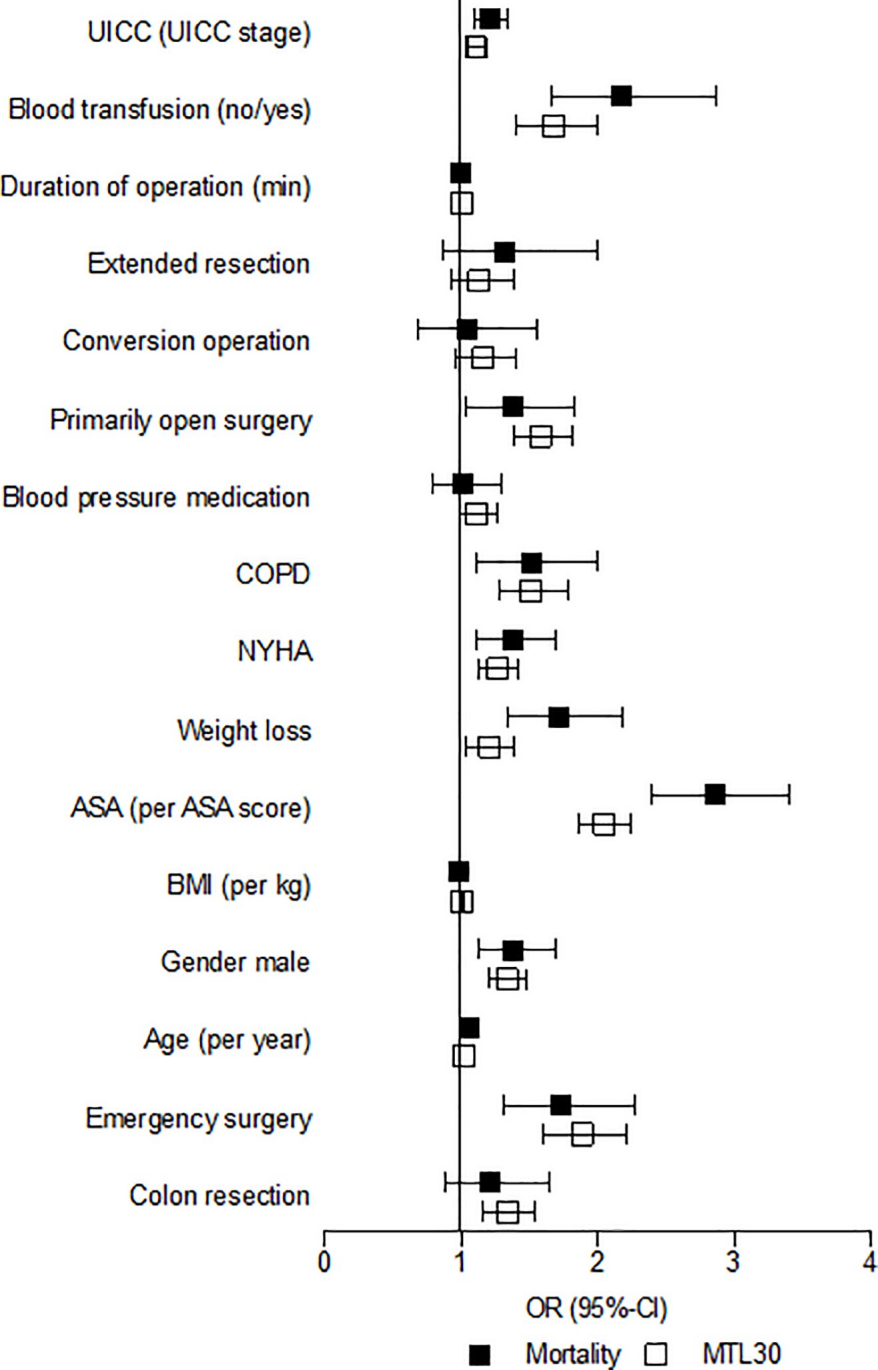
Adjusted Odds-ratio for risk factors.

The multivariable analysis for known patient-dependent factors as ASA or age as well as tumor-dependent factors as UICC-stage showed a significantly increased odds ratio (OR) for a positive MTL30. Similarly, the occurrence of postoperative complications increased the odds ratio for a positive MTL30. The 30-day lethality also showed a correlation with a number of risk factors and the occurrence of complications. But the 95% CI was smaller in all parameters arguing for a higher accuracy of the MTL30 (Table 5; Fig 1).

**Table 5:** Adjusted Odds-ratio for risk factors.

	MTL30			Mortality		
	Odds Ratio	95%-CI	p(chi)	Odds Ratio	95%-CI	p(chi)
Colon resection	1,3389	1,1606–1,5447	<0.001	1,21	0,88–1,65	0,24
Emergency surgery	1,8878	1,6081–2,2163	<0.001	1,73	1,31–2,28	<0.001
Age (per year)	1,0222	1,0165–1,0279	<0.001	1,06	1,05–1,07	<0.001
Gender male	1,3381	1,2054–1,4853	<0.001	1.38	1,13–1,69	0,002
BMI (per kg)	1,0139	1,0038–1,0241	0,007	0.99	0,97–1,02	0,77
ASA (per ASA score)	2,0459	1,8694–2,2391	<0.001	2,86	2,4–3,4	<0.001
Weight loss	1,2061	1,0459–1,3909	0,010	1,72	1,35–2,18	<0.001
NYHA	1,2714	1,1336–1,4260	<0.001	1,38	1,12–1,7	0,003
COPD	1,5151	1,2856–1,7856	<0.001	1,51	1,12–2,0	0,004
Blood pressure medication	1,1152	0,9861–1,2612	0,082	1,02	0,79–1,3	0,91
Primarily open surgery	1,5811	1,3834–1,8069	<0.001	1,38	1,04–1,83	0,025
Conversion operation	1,1639	0,9679–1,3996	0,11	1,04	0,69–1,56	0,85
Extended resection	1,1354	0,9302–1,3860	0,21	1,32	0,87–2,0	0,19
Duration of operation (min)	1,0027	1,0020–1,0034	<0.001	1,00	1.00–1.00	0,32
Blood transfusion (no/yes) V75	1,6794	1,4110–1,9988	<0.001	2,18	1,66–2,87	<0.001
UICC (UICC stage)	1,1097	1,0547–1,1676	<0.001	1,21	1,10–1,34	<0.001

## Discussion

Benchmarking is increasingly used within the healthcare sector as an approach to increase quality [19]. Nevertheless, valid assessment of surgical quality remains a major challenge [20].

Because morbidity and mortality decrease with the number of surgeries performed in hospitals [3, 4], “minimum quantities” have been recommended internationally. This has led to centralization in high volume centers [21, 22], but without necessarily improving surgical care. Indeed, it is the management of complications that appears to be better in high-volume hospitals. In addition, this volume-outcome relationship only applies to major interventions, since no volume-dependent differences have been shown for minor interventions [23]. A major problem with measuring the difference between surgical departments is that complications need to be documented in an unbiased manner, which necessitates an external audit. On the other hand, measured events need to be frequent enough to result in significant differences—particularly when cases are low. With the MTL30, we introduced a composite marker that allows a comparison between multiple hospitals across different levels of care and complexity of patient groups [12]. The MTL30 is based on the idea that there are no/minor complications if the patient can be released alive within 30 days of the operation. On the other hand, the operative/postoperative course must be regarded as conspicuous if the patient has died within 30 days, has been transferred to another acute care hospital, or is still in inpatient care after 30 days. Any serious medical intervention possesses a certain degree of morbidity and mortality, which explains why an MTL30 of 0% is extremely unlikely. The percentage of MTL30 positive patients is therefore dependent on the type of intervention performed (e.g. inguinal herniotomy vs. pancreatic resection), the patient's co-morbidity (e.g. ASA status), and the quality of the intervention. In addition, the index time-point of 30 days after operation was chosen for the MTL30 in accordance with the widely used 30-day mortality standard. Nevertheless, it can be adjusted to 7 or 14 days for other kinds of operations or other healthcare systems.

As a proof of concept, we showed for almost 20,000 datasets from the StuDoQ register that a positive MTL30 has a high correlation with existing risk factors of the patient, and strongly correlated with the occurrence of postoperative complications. As a composite indicator, the MTL30 is also more specific and sensitive than individual indicators.

Correctly determining surgical quality using QIs is a complex task [24]. On the one hand, markers should be used which are easy to collect, for example on the basis of accounting data, and which are difficult to falsify. This is indeed the case for the MTL30, since both the postoperative length of stay and the type of discharge from inpatient care (deceased, relocated, or discharged home/transferred to rehabilitation) are recorded in the accounting system. On the other hand, markers should be adjustable to ensure a fair comparison between hospitals. This was confirmed in the present study, where we used data from the StuDoQ register.

For a simple comparison between hospitals at different care levels, a global QI should ideally map all three pillars of quality to enable a fair comparison. If the outcome quality in terms of mortality is only considered, then the structural quality is overlooked. This includes e.g. the equipment of the hospital, the 24-hour preparedness for surgery, or the possibility of performing CT imaging. A low structural quality in a hospital therefore results in a high patient transfer rate, which is included in the recording of the "transfer" in the MTL30. In this way, it can be shown whether hospitals per se are appropriately equipped for the procedure performed. On the other hand, the length of stay (L) would be longer if hospitals were suitably equipped but are of worse quality due to disproportional complication rates.

Up to now, quality indicators are typically used to detect adverse events. By using the negative MTL30 as a quality indicator, we specifically tried to identify a “normal” postoperative course and could show a high degree of specificity (90%).

Determining significant differences between facilities depends on two basic parameters: The first is the number of cases carried out, and the second is the relative frequency of an "adverse" event. In order to show a significant difference between hospitals with, for example, 10% vs. 5% mortality, 185 interventions would be required [9] which increases to as many as 1, 000 cases in order to detect a statistically valid difference between 1% and 2% adverse events. In addition, mortality is a rare event overall, which explains why it is difficult to map a significant difference due to the small number of cases. Furthermore, mortality increasingly occurs after 30 days due to the continual improvement of intensive care medicine. For this reason, 90-day mortality is increasingly considered to be more appropriate [25]. However, these patients are still inpatient on the 30th day, and are therefore rated as conspicuous in the MTL30. Since the MTL30 subsumes several adverse events, and is therefore higher per se, the number of cases required to detect significant differences decreases. Furthermore, different operations can be clustered into groups of similar complexities to further increase the number of cases. In this way, a higher number of cases per hospital can be achieved, even with relatively rare interventions such as esophagus, stomach, and pancreatic resections.

One shortcoming of the MTL30 is that it does not detect minor changes within the postoperative course that do not prolong the in-patient care or lead to transfer to another unit (e.g. superficial side infection). Also, it does not differentiate between an eventful operative course from an eventful postoperative course. The major goal for further validation will be to determine the optimal cut-off timepoint for different operative procedures according to the nationally used healthcare system.

It has been shown previously that in addition to directly comparing between hospitals, self-evaluation leads to an increase in quality in individual hospitals, which is referred to as the Hawthorne effect [26]. The morbidity and mortality in participating hospitals decreased significantly due to the introduction of NSQIP [27]. As the MTL30 is easy to obtain and does not need extra documentation, it will give each individual hospital a quick and unbiased overview of where it stands in comparison with others.

In summary, measuring quality is a fundamental task in medicine. The MTL30 is an easily obtained and adjustable QI. A positive MTL30 shows high sensitivity for an eventful postoperative course, whereas a negative MTL30 has high specificity for an uneventful postoperative course.

## Supporting information

S1 Table Overview of patient characteristic. (DOCX)
