# Supplementary material for: Validation of MTL30 as a quality indicator for colorectal surgery
Source: PLoS One. 2020 Aug 28;15(8):e0238473. doi: 10.1371/journal.pone.0238473 (PMC7454590; doi:10.1371/journal.pone.0238473)
Supplement: S1 Table — (DOCX) [file pone.0238473.s001.docx]

Supplement Table 1 – overview of patient characterisitc

|  |  |  | n | % |
| --- | --- | --- | --- | --- |
| Number of patients |  |  | 19646 | 100 |
| Gender |  | male | 10973 | 55.85 |
|  |  | female | 8673 | 44.15 |
| Age |  | 18 - 59 | 4024 | 20.48 |
|  |  | 60 - 69 | 4645 | 23.64 |
|  |  | 70 - 79 | 6981 | 35.53 |
|  |  | 80 - 100 | 3973 | 20.22 |
|  |  | *not known* | 23 | – |
|  |  | Ø 69.5 ± 11.9 |  |  |
| BMI [kg×m^-2^] |  | < 18 | 279 | 1.42 |
|  |  | 18 – 25 | 7667 | 39.04 |
|  |  | 25 – 30 | 7451 | 37.94 |
|  |  | 30 – 35 | 3099 | 15.78 |
|  |  | 35 – 40 | 831 | 4.23 |
|  |  | > 40 | 314 | 1.61 |
| ASA |  | 1, normal | 1678 | 8.54 |
|  |  | 2 | 9413 | 47.92 |
|  |  | 3 | 7977 | 40.61 |
|  |  | 4 | 564 | 2.87 |
|  |  | 5 | 13 | 0.07 |
|  |  | *not known* | 1 | – |
| ECOC status |  | none | 17780 | 90.50 |
|  |  | partially | 1621 | 8.25 |
|  |  | full | 245 | 1.25 |
| Weight loss |  | no | 17365 | 88.52 |
|  |  | yes | 2252 | 11.48 |
|  |  | *not known* | 29 | – |
| UICC |  | I | 5373 | 27.54 |
|  |  | II | 6093 | 31.23 |
|  |  | III | 5500 | 28.20 |
|  |  | IV | 2541 | 13.03 |
|  |  | *not known* | *139* | *–* |
| Diabetes mellitus |  | no | 15698 | 79.90 |
|  |  | NIDDM | 2614 | 13.31 |
|  |  | IDDM | 1334 | 6.79 |
| Cerebrovascular event |  | no | 18223 | 92.76 |
|  |  | yes, w/o deficit | 869 | 4.42 |
|  |  | yes, with deficit | 554 | 2.82 |
| CAD |  | no | 16130 | 82.10 |
|  |  | yes | 3516 | 17.90 |
| NYHA |  | no | 15587 | 79.34 |
|  |  | I | 972 | 4.95 |
|  |  | II | 1665 | 8.48 |
|  |  | III | 830 | 4.22 |
|  |  | IV | 84 | 0.43 |
|  |  | *not defined* | 508 | 2.59 |
| Dialysis |  | no | 19521 | 99.36 |
|  |  | yes | 125 | 0.64 |
| COPD |  | no | 18512 | 94.23 |
|  |  | yes | 1134 | 5.77 |
| PAOD |  | no | 19117 | 97.31 |
|  |  | yes | 529 | 2.69 |
| Blood pressure medication |  | no | 7932 | 40.37 |
|  |  | yes | 11714 | 59.63 |
| Radio-/Chemotherapy |  | no | 17063 | 86.85 |
|  |  | Radiotherapy | 181 | 0.92 |
|  |  | Chemotherapy | 190 | 0.97 |
|  |  | combined | 2212 | 11.26 |
| Corticoids |  | no | 19403 | 98.76 |
|  |  | yes | 243 | 1.24 |
| Immunosuppressants |  | no | 19498 | 99.25 |
|  |  | yes | 148 | 0.75 |
| Anticoagulants |  | no | 15971 | 81.29 |
|  |  | yes | 3675 | 18.71 |
| Alcohol abuse |  | no | 18886 | 96.18 |
|  |  | yes | 751 | 3.82 |
|  |  | *not known* | 9 | – |
| Child-Score |  | no liver cirrhosis | 19183 | 97.99 |
|  |  | Child A | 171 | 0.87 |
|  |  | Child B | 57 | 0.29 |
|  |  | Child C | 24 | 0.12 |
|  |  | liver cirrhosis, C unknown | 142 | 0.73 |
|  |  | *not known* | 69 | – |
| MTL30 |  | normal | 17762 | 90,41 |
|  |  | divergent | 1884 | 9,59 |
